# Structure–Activity Relationship of 5-mer Catalytides, GSGYR and RYGSG

**DOI:** 10.3390/biom12121766

**Published:** 2022-11-27

**Authors:** Rina Nakamura, Toshifumi Akizawa, Motomi Konishi

**Affiliations:** 1 Laboratory of Pharmacology, School of Medicine, Koch University, Nankoku 783-0047, Japan; 2 O-Force Co., Ltd., 3454 Irino Kuroshio-cho, Hata-gun, Kochi 789-1931, Japan; 3 Department of Integrative Pharmaceutical Sciences, Faculty of Pharmaceutical Sciences, Setsunan University, Hirakata 573-0101, Japan

**Keywords:** Catalytide, in silico, Alzheimer’s disease, amyloid-β, hydrolytic peptide, structure–activity relationship

## Abstract

We recently discovered JAL-TA9 (YKGSGFRMI), a short hydrolytic peptide that we termed a Catalytide. The catalytic center of JAL-TA9 was modeled using MM2 and MMFF94 parameters and identified as GSGFR. Additionally, a structure–activity relationship study showed that GSGYR cleaved Aβ11-29. Here, we developed a novel Catalytide in silico. Molecular dynamics simulations of GSGYR and RYGSG using MM2 and MMFF94 parameters suggested that both peptides may form catalytic triads and oxyanion holes. The hydrolytic potency of RYGSG was five times higher than that of GSGYR. Moreover, both peptides showed three common cleavage positions for Aβ11-29; namely, L17-V18, V18-F19, and E22-D23. The aggregation ratio analyzed by the thioflavin-T assay correlated well with proteolytic activity, suggesting that the aggregation of Aβ11-29 was suppressed by the cleavage reaction. Docking simulations with the carbonyl carbon of L17 or the carbonyl carbon of E22 in Aβ11-29 were conducted using the secondary structures of GSGYR and RYGSG. The distance between the hydroxyl group of serine and the carbonyl carbon of the two cleavage sites proved that RYGSG was closer to Aβ11-29 than to GSGYR. This study demonstrated that Catalytides are useful for understanding structure–activity relationships.

## 1. Introduction

We previously reported the proteolytic activity of the 9-mer peptide JAL-TA9 (YKGSGFRMI) derived from the Box A region of Tob1, a highly conserved region in the Tob/BTG protein family. Because small peptides possessing proteolytic activity were not found before this study, we used the general name Catalytide for hydrolase peptides such as JAL-TA9 [1].

JAL-TA9 shows potent proteolytic activity against amyloid-beta (Aβ) peptide fragments. Additionally, it can cleave both the soluble and solid forms of Aβ42, causing Alzheimer’s disease (AD) in the central region of Aβ42 [2]. AD is the most common age-related neurodegenerative disorder, and is caused by the aggregation and accumulation of Aβ42 [3,4]. Therefore, Aβ42 is predicted to be a potentially effective target for drug therapy. Furthermore, these data indicate that Catalytides may represent a potent Alzheimer’s drug with a novel mechanism compared toβ- or γ-secretases inhibitors, or inhibitors of Aβ42 oligomerization, such as aducanumab [5,6,7].

The secondary structure of JAL-TA9, determined using NMR and computer modeling, indicated that JAL-TA9 consisted of a catalytic triad and an oxyanion hole in its compact structure. Additionally, systematic studies of Catalytides have identified that the size of the 5-mer peptide, GSGFR, is the minimum size for the proteolytic activity of JAL-TA9 [1,8].

The Box A region is a highly conserved homology domain of Tob/BTG family proteins, which comprise Tob1, Tob2, BTG1, BTG2, BTG3/ANA, and BTG4. Although there are many reports on the function of the Tob/BTG family, such as antiproliferative activity in a variety of cell types and the regulation of tumorigenesis, the role of the Box A region remains unclear [9,10,11,12,13,14,15]. Our previous study showed that 5-mer synthetic peptides derived from the Box A region of the Tob/BTG family, GQAYR (BTG3) and GQAFR (BTG4), showed higher activity than GSGYR (BTG1 and 2) and GSGFR (Tob 1 and 2) [8], indicating that 5-mer Catalytides are attractive candidates for developing peptide drugs as a new strategy for treating AD.

Structure-based peptide or protein designs that modulate target substrates in silico have recently been developed. In this study, two types of 5-mer synthetic peptides, a GSGYR-derived Box A region of the BTG1 protein and reverse sequence (RYGSG), were used to elucidate the relationship between the stereo-structure and proteolytic activity of the 5-mer peptide using a personal computer (PC) through molecular modeling with MM2 and MMFF94 parameters. Our study revealed that RYGSG forms an oxyanion hole and a catalytic triad similar to GSGYR. Furthermore, RYGSG showed five-fold stronger catalytic activity than GSGYR. We demonstrated the utility of PC-based modeling for creating a novel Catalytide in silico. This 5-mer peptide is a valuable seed for creating potent proteolytic peptides against Aβ.

## 2. Materials and Methods

### 2.1. Peptide Preparation

Peptides were prepared and their proteolytic activity was measured as described in our previous study [1,2]. The peptides were synthesized (model 433A, Applied Biosystems, Foster City, CA, USA.; 0.1 mmol scale with preloaded resin). After deprotection according to the manufacturer’s protocol, each peptide was purified using reverse-phase preparative high-performance liquid chromatography (RP-HPLC), (Capcell Pak C18 column, SG, 15 mm i.d. 250 mm; OSAKA SODA Co., Ltd., Osaka, Japan) and the molecular weights of the peptides were confirmed by analytical HPLC (CapcellPak C18 column, MGII, 4.6 mm i.d. 150 mm; OSAKA SODA Co., Ltd., Osaka, Japan). Each purified peptide was characterized by electrospray ionization mass spectrometry using (ESI-MS) a Qstar Elite Hybrid LC–MS/MS system [1,2].

### 2.2. Stereo-Structure Analysis

The computer modeling was performed using the Software CSC Chem3D UltraTM (version 15.1., PerkinElmer, Waltham, MA, USA). The solvent radius was 1.4 Å, which is the value for water. The structures of 5-mer peptides were calculated under the conditions described below. Initially, all peptide bond and dihedral angles were set to 180°. Subsequently, the six atoms comprising the peptide bond were arranged in one plane, and the bond lengths were set. We then conducted calculations using structural optimization and energy minimization according to MM2 and MMFF94 parameters (bond length, bond angles, dihedral angles, dipole moments, and van der Waals values) [1,16].

### 2.3. Proteolytic Activities

GSGYR and RYGSG (final concentration 0.2 mM) were individually incubated with the peptide fragment Aβ11-29 from Aβ42 (final concentration 0.05 mM) in the presence of HSA (final concentration 0.025% *w*/*v*) in phosphate-buffered saline (PBS; pH 7.4) at 37 °C. Each 10 μL reaction mixture was analyzed in a time-dependent manner using the analytical HPLC system described above. For peak collection, 20 μL of the reaction mixture was loaded into the HPLC system, and the peak fractions were monitored at 220 nm and collected in microtubes (Eppendorf safe-lock tubes, 1.5 mL).

After lyophilization, an appropriate quantity of 36% CH_3_CN containing 0.1% HCOOH was added with stirring using an automatic mixer, as determined from the chromatographic peak heights. The cleavage sites were determined by ESI-MS using the flow injection method with 70% CH_3_CN containing 0.1% HCOOH on a Qstar Hybrid LC-MS/MS system, and the flow rate was set to 0.1 mL/min [1].

### 2.4. Thioflavin-T (ThT) Assay

A ThT assay was performed to evaluate the aggregation of Aβ11-29. Aβ11-29 (final concentration 100 μM) was incubated with GSGYR and RYGSG (final concentration 100 μM) in PBS buffer (pH 7.4). The ThT solution (final concentration 100 μM) was added immediately before measurement. The ThT signal was monitored by excitation at 444 nm and fluorescence emission was measured at 480 nm for 10 s using a cell imaging multimode reader (Cytaion 5; BioTek, Santa Clara, CA, USA).

### 2.5. Low-Vacuum Scanning Electron Microscopy (LV-SEM)

Aβ11-29 (final concentration 100 µM) was incubated with GSGYR and RYGSG (final concentration 100 μM) in PBS (pH 7.4) at 37 °C. After 1 week, the reaction solution was centrifuged at 9000 rpm for 5 min, the supernatant was removed, and 200 µL of MilliQ water was added, mixed, and centrifuged at 9000 rpm for 5 min three times to remove the salt present in the reaction mixture. Subsequently, 200 µL of 1% phosphotungstic acid was added, and was washed with MilliQ water after 1 min. After a final wash with MilliQ water, the reaction solution was dried on an electrically conductive tape (NISSHIN EM Co., Ltd., Tokyo, Japan) and observed using LV-SEM (Miniscope^®^TM3030Plus, HITACHI Co., Tokyo, Japan) [17]. LV-SEM was performed under an accelerating voltage of 5.3 kV, vacuum degree of 30 Pa, and observation magnification of 1000×.

### 2.6. Docking Simulation

All the simulations were performed using the Software CSC Chem3D UltraTM (version 15.1., PerkinElmer, Waltham, MA, USA) and docking simulations were performed using Auto Dock Interface (version 15.1., Center for Computational Structural Biology, La Jolla, CA, USA). The solvent radius was 1.4 Å, which is the value for water. The obtained NMR structure of Aβ11-29 (PDB ID:1QXC) had a coiled conformation and 5-mer peptides were obtained from stereo-structure analysis. The docking parameters were as follows: grid map with 0° set to X, Y, and Z; the grid spacing was 0.5 Å, a population of 50 individuals, 27,000 generations, 250,000 energy evaluations, and 100 docking trials. This procedure was repeated ten times. After the calculations, we selected the best simulated binding energies and compared the locations of the 5-mer peptides on Aβ11-29.

### 2.7. Statistical Analysis

All the data are expressed as mean ± standard error of the mean (SEM). The statistical significance of the differences between experimental groups was measured using the Student’s t-test. The differences were considered significant at a *p* value of 0.05.

## 3. Results and Discussions

### 3.1. Stereo-Structure Analysis of 5-mer Peptides

**Figure 1:**
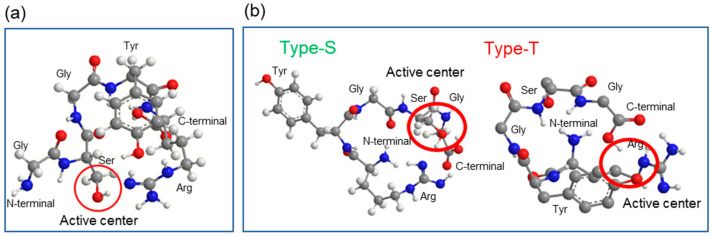
Computer modeling using MM2 and MMFF94 parameters of (**a**) GSGYR and (**b**) RYGSG. Hydrogen atom: white ball; carbon atom: gray ball; oxygen atom: red ball; nitrogen atom: blue ball. Red circle indicates active center.

Previously, we revealed that GSGFR is a serine protease whose active center is Ser, and that its single amino acid substitution, GSGYR, also exhibits proteolytic activity. Therefore, we first performed molecular dynamics (stereo-structure) simulations of two types of 5-mer peptides, GSGYR and its reverse sequence RYGSG, using MM2 and MMFF94 parameters. The predicted structures that were thought to exhibit enzymatic activity were shown in Figure 1. Stereo-structure analysis of GSGYR showed that the carbonyl oxygen at the C-terminal, base form (guanidyl group) of arginine (Arg), and hydroxyl group of serine (Ser) interact with each other and constitute the active center (Figure 1a).

**Figure 2:**
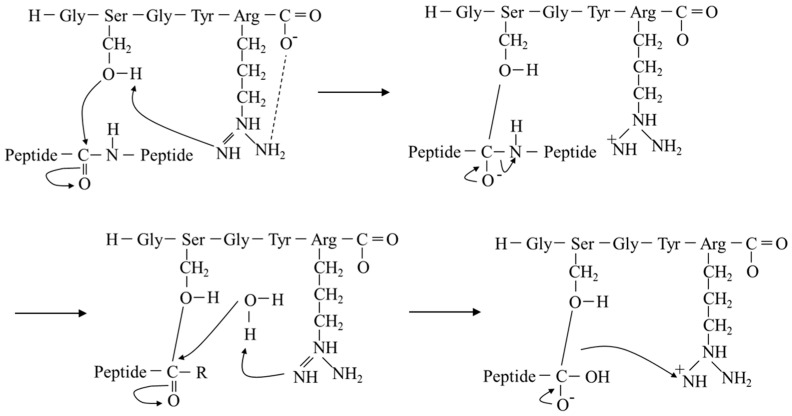
Proposed cleavage mechanism of GSGYR.

Based on these results, we hypothesized a cleavage mechanism for GSGYR (Figure 2). The nitrogen atom of the guanidyl group first abstracts a proton from the serine hydroxyl group. It then produces an oxyanion that acts as a nucleophile and attacks substrate peptide bonds. Subsequently, the Arg side chain donates a proton to the substrate amide nitrogen. Finally, water attacks the ester bond between the substrate and Ser (Figure 2). This hypothesis satisfies the generally accepted chemical mechanism of serine protease catalysis [18].

Two different structures, Type-S and Type-T, were obtained for the stereo structure of RYGSG, the reverse sequence of GSGYR (Figure 1b). In the case of Type-S, the positional relation of the three functional groups in the catalytic triad was similar to that of GSGYR, and Ser was thought to be the active center. In Type-T, the hydroxyl group of tyrosine (Tyr), rather than the hydroxyl group of Ser, was speculated to attack the carbonyl of the peptide substrate. Moreover, this reaction may be assisted by Arg as a general base. These results suggest that RYGSG could form two types of active structures, Type-S and Type-T, which could show proteolytic activity similar to that of GSGYR. Thus, we used GSGYR and RYGSG for subsequent experiments.

### 3.2. Proteolytic Activity of GSGYR and RYGSG Peptides

**Figure 3:**
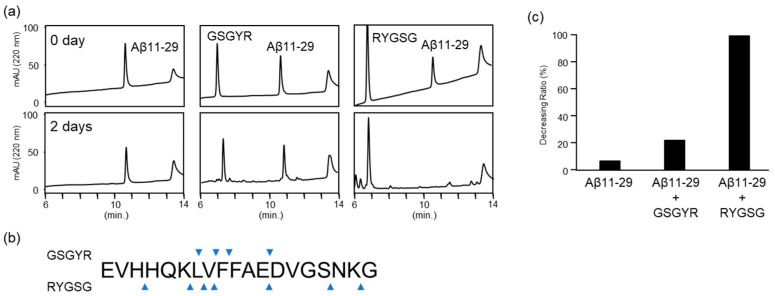
Cleavage reaction of Aβ11-29 by GSGYR and RYGSG. (**a**) Time-dependent analysis of the reaction mixture of Aβ11-29 alone (left), and with GSGYR (middle) and RYGSG (right) after 0 and 2 days. (**b**) The blue triangle indicates cleavage sites on Aβ11-29. (**c**) Decreasing ratio of Aβ11-29. The decreasing ratio was calculated by estimating the peak height of the chromatogram on day 0 and day 2.

We evaluated the proteolytic activity of GSGYR and RYGSG against Aβ11-29, which contains the intermediate region that is nucleated for the aggregation of Aβ42. Therefore, analyzing the strength of the cleavage activity against Aβ11-29 and the three-dimensional structure may pave the way for a basic clinical trial to create an enzyme peptide with a higher activity against Aβ42. Aβ11-29 alone did not show any changes during the 2 days of incubation (Figure 3a). In contrast, after 2 days of co-incubation with GSGYR, the relative intensities of the peaks corresponding to GSGYR and Aβ11-29 decreased slightly compared to day 0, and several small peaks appeared (Figure 3a). These results were similar to those of our previous study, which indicated the cleavage of Aβ11-29 by GSGYR. In the case of RYGSG, Aβ11-29 disappeared after 2 days of incubation, and new peaks appeared (Figure 3a).

Subsequently, we determined the cleavage site of Aβ11-29. Twenty microliters of the reaction mixture were analyzed via analytical HPLC. All the peaks appearing on day 2 were collected by monitoring at 220 nm and the cleavage site was determined by ESI-MS. In the reaction mixtures of Aβ11-29 and RYGSG, peaks A1–A5 and R1–R2 were identified as derivatives of Aβ11-29 and RYGSG, respectively (Figure 3b and Figure S1). Seven cleavage sites were identified in RYGSG (Figure 3b). The cleavage sites of Aβ11-29 for GSGYR were also analyzed in the same manner (Figure 3b and Figure S2). There was no cleavage site specificity, and both peptides showed three common cleavage positions: L17-V18, V18-F19, and E22-D23 (Figure 3b). The differences in the cleavage site were likely caused by the stereo-structures of GSGYR and RYGSG. In addition, MS analysis confirmed the presence of peptide fragments derived from GSGYR and RYGSG (Figures S1 and S2). This suggests that the decrease in peptide peak height in the reaction mixture was due to the autolysis of GSGYR and RYGSG. We calculated the decreasing ratio of Aβ11-29 based on peak heights on days 0 and 2. Based on the decreasing ratio, the proteolytic activity of RYGSG against Aβ11-29 was found to be five times that of GSGYR (Figure 3c).

### 3.3. Thioflavin (ThT) Assay

**Figure 4:**
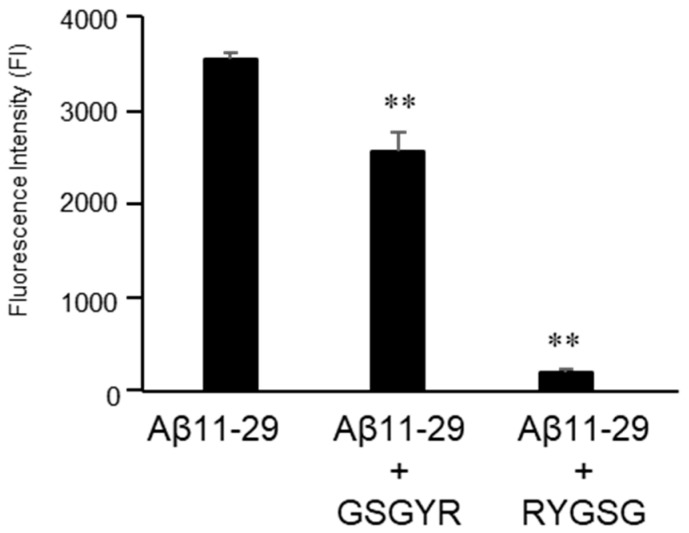
Effect of GSGYR and RYGSG on aggregation of Aβ11-29. Data are presented as mean ± SEM. *n* = 3. ** indicates *p* < 0.01 versus Aβ11-29.

The effects of GSGYR and RYGSG on the aggregation of Aβ11-29 (final concentration 0.1 mM) were analyzed using the ThT assay at 37 °C in PBS (Figure 4). The fluorescence intensity increased after 5 days in the presence of Aβ11-29 without the addition of 5-mer peptides. This indicated that Aβ11-29 has the potential to aggregate. However, when co-incubated with GSGYR or RYGSG at a final concentration of 0.1 mM, the fluorescence intensity was significantly reduced compared with Aβ11-29 alone, with 28% and 95% suppression, respectively (Figure 4). The aggregation of Aβ11-29 was probably suppressed by the proteolytic activity of GSGYR and RYGSG.

### 3.4. Scanning Electron Microscopy (SEM)

**Figure 5:**
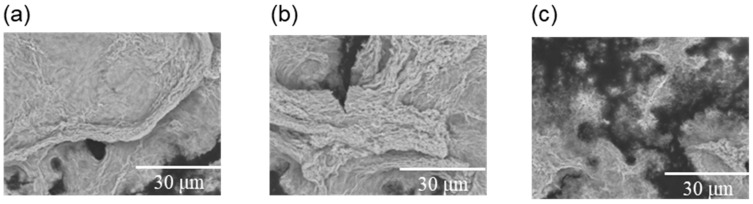
Scanning electron microscopy (SEM). (**a**) Aβ11-29, (**b**) Aβ11-29 co-incubate GSGYR, (**c**) Aβ11-29 co-incubate RYGSG.

We investigated the effect of GSGYR and RYGSG on amyloid formation by Aβ11-29 by observing its shape using SEM. When Aβ11-29 alone was incubated in PBS at 37 °C for 5 days, thick fibrils and a cluster of thin fibrils were observed (Figure 5a). Although fibrils were also observed in the presence of GSGYR (final concentration 0.1 mM), many were slightly finer than those of Aβ11-29 alone (Figure 5b). Furthermore, fibrils were rarely observed in the co-incubation with RYGSG; instead, short, thin fibrils were observed (Figure 5c), indicating a more potent inhibition of fibrillization of Aβ11-29 by RYGSG than by GSGYR. These data agree with the ThT results shown in Figure 4, which indicate the inhibitory effects of GSGYR and RYGSG on Aβ11-29 aggregation.

### 3.5. Docking Simulation

**Figure 6:**
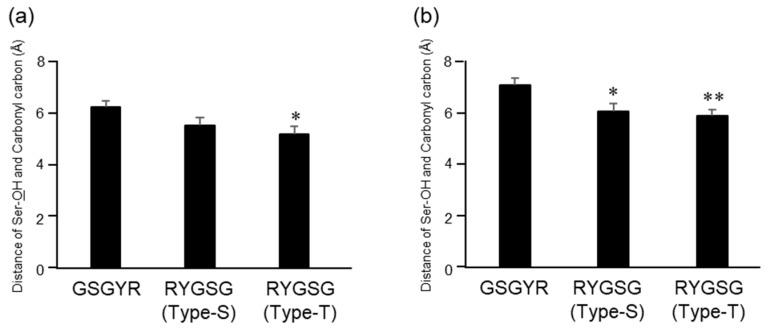
Comparison of the distance between the Ser-OH of each 5-mer peptide and the carbonyl carbon of the cleavage site. (**a**) Carbonyl carbon of 17Leu, (**b**) carbonyl carbon of 22Glu. Data are presented as mean ± SEM. *n* = 10. * indicates *p* < 0.05 and ** indicates *p* < 0.01 versus Aβ11-29.

Docking simulation was performed between Aβ11-29, and RYGSG or GSGYR, the structures of which were obtained from Figure 1. The common cleavage points between 17Leu-18Val and 22Glu-23Asp are shown in Figure 3. We performed calculations centered on the carbonyl carbons of the two peptide bonds. First, a docking simulation was performed between the carbonyl carbon of L17 in Aβ11-29 and GSGYR. The distance between the hydroxyl group of Ser in GSGYR and the carbonyl carbon of L17 was 6.3 Å, and the free energy was −2296.21 kcal/mol (Table S1a and Figure 6a).

Subsequently, we performed a docking simulation between the carbonyl carbon of L17 in Aβ11-29 and RYGSG (Type-S and Type-T). In the case of Type-S, the distance of the carbonyl carbon of L17 in Aβ11-29, and the hydroxyl group of Ser and Tyr, were 5.59 and 6.66 Å, respectively; and the free energy was −2280.96 kcal/mol (Table S1a and Figure 6a). The distance between the carbonyl carbon of L17 in Aβ11-29, and the hydroxyl group of Ser and Tyr in Type-T, were 5.26 and 6.51 Å, respectively; and the free energy was −2310.88 kcal/mol (Table S1a and Figure 6a). Therefore, in both types, the hydroxyl group of Ser was closer to the carbonyl carbon of L17 in Aβ11-29 than the hydroxyl group of Tyr.

The docking simulation between the carbonyl carbon of E22 in Aβ11-29, and GSGYR or RYGSG (Type-S and Type-T), was performed in the same manner. In Type-S, the distance between the carbonyl carbon of E22 in Aβ11-29, and the hydroxyl group of Ser and Tyr, were 6.05 and 7.44 Å, respectively; and the free energy was −2549.20 kcal/mol (Table S1b and Figure 6b). In Type-T, the distance between the carbonyl carbon of E22 in Aβ11-29, and the hydroxyl group of Ser or Tyr, was 5.89 or 6.95 Å, respectively; and the free energy was −2555.57 kcal/mol (Table S1b and Figure 6b). Therefore, in both types, the hydroxyl group of Ser was closer to the carbonyl carbon of L17 than the hydroxyl group of Tyr.

The distance between the hydroxyl group of Ser in RYGSG and the carbonyl carbon of the two cleavage sites was significantly shorter than that in GSGYR (Figure 6). These data support the finding that the catalytic activity of RYGSG was five times stronger than that of GSGYR. The docking simulations of GSGYR and RYGSG (Type-S and Type-T), with the carbonyl carbons of 17Leu and 22Glu are shown in Figure S3.

Although stereo-structural analysis using MM2 suggested that the Tyr of RYGSG was the active center, the docking simulation showed a structure wherein Ser of RYGSG was considered to be the active center. It was thought that the conformation of RYGSG changes in the presence of a substrate. These data suggest that both stereo-structural analysis with MM2 and docking simulation with a substrate is important for the stereo-structural analysis of Catalytides.

## 4. Conclusions

In this study, we show that Aβ is a viable therapeutic target for drug therapy for AD [5,6,16]. Our previous study reported the proteolytic activity of the synthetic 5-mer peptide GSGFR toward Aβ. Here, we evaluated the relationship between the structure and proteolytic activity of GSGYR and RYGSG using computer modeling and docking simulations of Aβ11-29. Computer modeling showed that RYGSG formed a compact secondary structure containing the catalytic triad and oxyanion hole required for serine protease-like activity. The proteolytic activity of RYGSG was five-fold higher than that of GSGYR. Docking simulations of Aβ11-29, and GSGYR or RYGSG showed that the Ser-OH of RYGSG was significantly shorter than that of GSGYR. Therefore, a combination of in silico modeling and docking simulation is a helpful method for creating new peptide drugs for AD treatment. Furthermore, this method can be used to search for peptides that are effective against other neurotoxic diseases caused by the oligomerization/aggregation of proteins, such as tau [19], α-synuclein [20,21,22,23], SOD1 [24], TDP43 [25,26], and prions [27,28,29,30,31].

## 5. Patents

T. Yamamoto and T. Akizawa, 2016, NOVEL HYDROLASE-LIKE PEPTIDE AND ITS USE, US62/275,599.

## Data Availability

The data and materials in this article are available from the corresponding authors on reasonable request.

## Supplementary Materials

The following supporting information can be downloaded at: https://www.mdpi.com/article/10.3390/biom12121766/s1, Figure S1: Identification of cleavage sites of Aβ11-29 by RYGSG; Figure S2: Identification of cleavage sites of Aβ11-29 by GSGYR; Figure S3: Representative images of ten docking simulations; Table S1: Docking simulation.
