# Supplementary material for: Structure–Activity Relationship of 5-mer Catalytides, GSGYR and RYGSG
Source: Biomolecules. 2022 Nov 27;12(12):1766. doi: 10.3390/biom12121766 (PMC9775622; doi:10.3390/biom12121766)
Supplement: Supplementary file 1 [file biomolecules-12-01766-s001.zip › biomolecules-2004814-supplementary.pptx]

## Slide 1
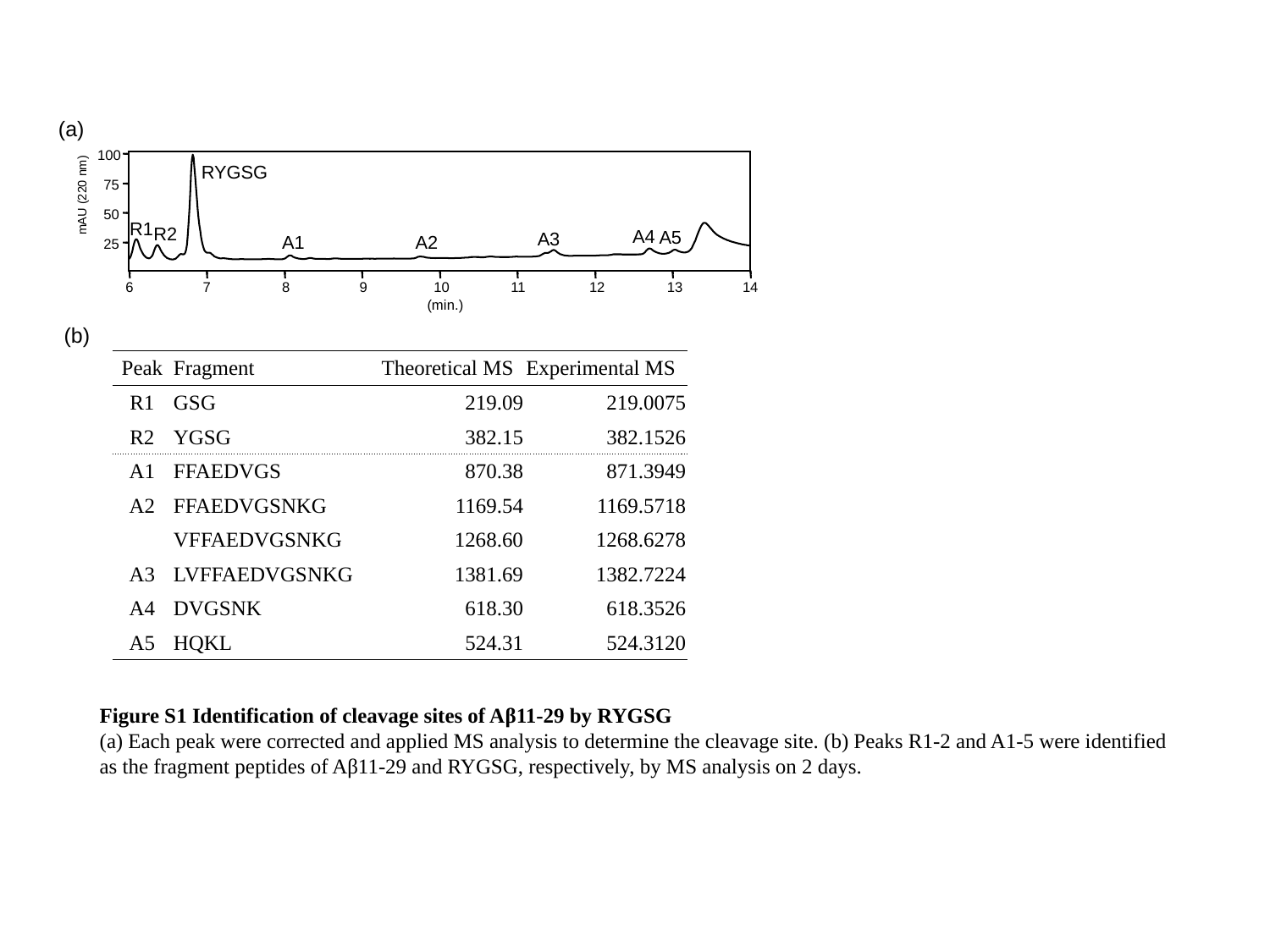

(a)
100
RYGSG
75
mAU (220 nm)
50
R1
R2
A4
A5
A3
A1
A2
25
6
7
8
9
10
11
12
13
14
(min.)
(b)
| Peak | Fragment | Theoretical MS | Experimental MS |
| --- | --- | --- | --- |
| R1 | GSG | 219.09 | 219.0075 |
| R2 | YGSG | 382.15 | 382.1526 |
| A1 | FFAEDVGS | 870.38 | 871.3949 |
| A2 | FFAEDVGSNKG | 1169.54 | 1169.5718 |
| | VFFAEDVGSNKG | 1268.60 | 1268.6278 |
| A3 | LVFFAEDVGSNKG | 1381.69 | 1382.7224 |
| A4 | DVGSNK | 618.30 | 618.3526 |
| A5 | HQKL | 524.31 | 524.3120 |
Figure S1 Identification of cleavage sites of Aβ11-29 by RYGSG
(a) Each peak were corrected and applied MS analysis to determine the cleavage site. (b) Peaks R1-2 and A1-5 were identified as the fragment peptides of Aβ11-29 and RYGSG, respectively, by MS analysis on 2 days.

## Slide 2
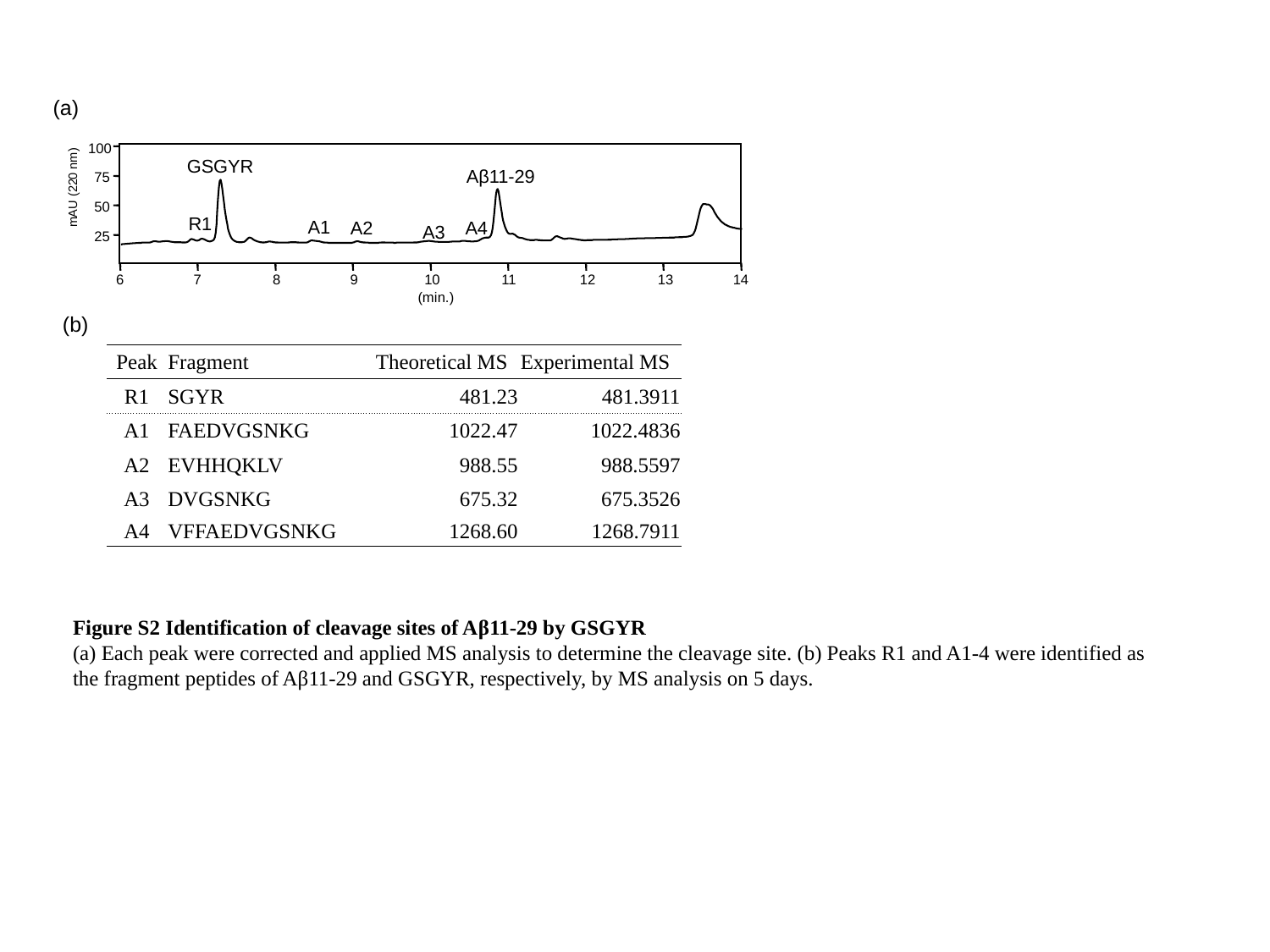

(a)
100
GSGYR
Aβ11-29
75
mAU (220 nm)
50
R1
A1
A4
A2
A3
25
6
7
8
9
10
11
12
13
14
(min.)
(b)
| Peak | Fragment | Theoretical MS | Experimental MS |
| --- | --- | --- | --- |
| R1 | SGYR | 481.23 | 481.3911 |
| A1 | FAEDVGSNKG | 1022.47 | 1022.4836 |
| A2 | EVHHQKLV | 988.55 | 988.5597 |
| A3 | DVGSNKG | 675.32 | 675.3526 |
| A4 | VFFAEDVGSNKG | 1268.60 | 1268.7911 |
Figure S2 Identification of cleavage sites of Aβ11-29 by GSGYR
(a) Each peak were corrected and applied MS analysis to determine the cleavage site. (b) Peaks R1 and A1-4 were identified as the fragment peptides of Aβ11-29 and GSGYR, respectively, by MS analysis on 5 days.

## Slide 3
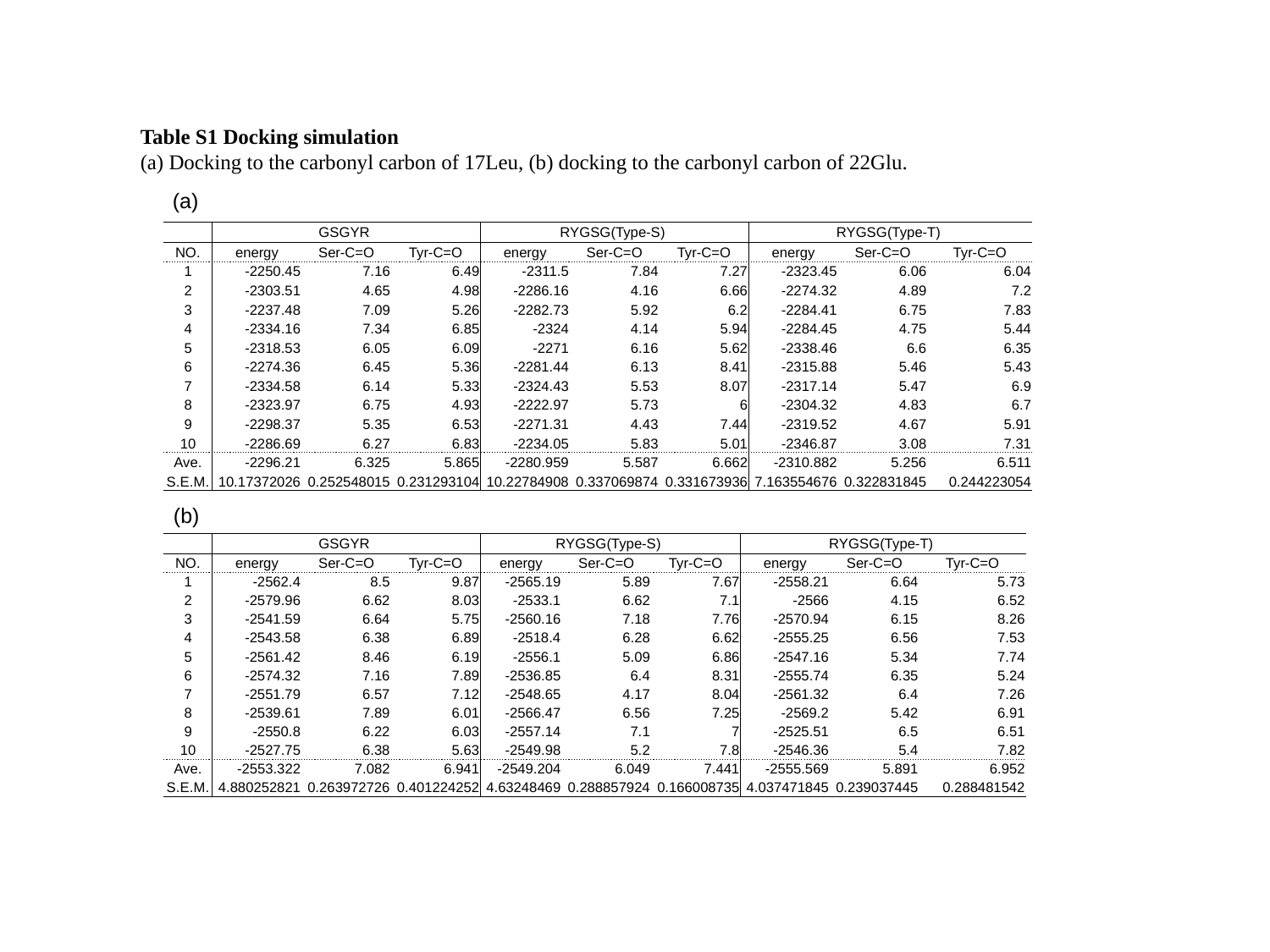

Table S1 Docking simulation
(a) Docking to the carbonyl carbon of 17Leu, (b) docking to the carbonyl carbon of 22Glu.
(a)
| | GSGYR | | | RYGSG(Type-S) | | | RYGSG(Type-T) | | |
| --- | --- | --- | --- | --- | --- | --- | --- | --- | --- |
| NO. | energy | Ser-C=O | Tyr-C=O | energy | Ser-C=O | Tyr-C=O | energy | Ser-C=O | Tyr-C=O |
| 1 | -2250.45 | 7.16 | 6.49 | -2311.5 | 7.84 | 7.27 | -2323.45 | 6.06 | 6.04 |
| 2 | -2303.51 | 4.65 | 4.98 | -2286.16 | 4.16 | 6.66 | -2274.32 | 4.89 | 7.2 |
| 3 | -2237.48 | 7.09 | 5.26 | -2282.73 | 5.92 | 6.2 | -2284.41 | 6.75 | 7.83 |
| 4 | -2334.16 | 7.34 | 6.85 | -2324 | 4.14 | 5.94 | -2284.45 | 4.75 | 5.44 |
| 5 | -2318.53 | 6.05 | 6.09 | -2271 | 6.16 | 5.62 | -2338.46 | 6.6 | 6.35 |
| 6 | -2274.36 | 6.45 | 5.36 | -2281.44 | 6.13 | 8.41 | -2315.88 | 5.46 | 5.43 |
| 7 | -2334.58 | 6.14 | 5.33 | -2324.43 | 5.53 | 8.07 | -2317.14 | 5.47 | 6.9 |
| 8 | -2323.97 | 6.75 | 4.93 | -2222.97 | 5.73 | 6 | -2304.32 | 4.83 | 6.7 |
| 9 | -2298.37 | 5.35 | 6.53 | -2271.31 | 4.43 | 7.44 | -2319.52 | 4.67 | 5.91 |
| 10 | -2286.69 | 6.27 | 6.83 | -2234.05 | 5.83 | 5.01 | -2346.87 | 3.08 | 7.31 |
| Ave. | -2296.21 | 6.325 | 5.865 | -2280.959 | 5.587 | 6.662 | -2310.882 | 5.256 | 6.511 |
| S.E.M. | 10.17372026 | 0.252548015 | 0.231293104 | 10.22784908 | 0.337069874 | 0.331673936 | 7.163554676 | 0.322831845 | 0.244223054 |
(b)
| | GSGYR | | | RYGSG(Type-S) | | | RYGSG(Type-T) | | |
| --- | --- | --- | --- | --- | --- | --- | --- | --- | --- |
| NO. | energy | Ser-C=O | Tyr-C=O | energy | Ser-C=O | Tyr-C=O | energy | Ser-C=O | Tyr-C=O |
| 1 | -2562.4 | 8.5 | 9.87 | -2565.19 | 5.89 | 7.67 | -2558.21 | 6.64 | 5.73 |
| 2 | -2579.96 | 6.62 | 8.03 | -2533.1 | 6.62 | 7.1 | -2566 | 4.15 | 6.52 |
| 3 | -2541.59 | 6.64 | 5.75 | -2560.16 | 7.18 | 7.76 | -2570.94 | 6.15 | 8.26 |
| 4 | -2543.58 | 6.38 | 6.89 | -2518.4 | 6.28 | 6.62 | -2555.25 | 6.56 | 7.53 |
| 5 | -2561.42 | 8.46 | 6.19 | -2556.1 | 5.09 | 6.86 | -2547.16 | 5.34 | 7.74 |
| 6 | -2574.32 | 7.16 | 7.89 | -2536.85 | 6.4 | 8.31 | -2555.74 | 6.35 | 5.24 |
| 7 | -2551.79 | 6.57 | 7.12 | -2548.65 | 4.17 | 8.04 | -2561.32 | 6.4 | 7.26 |
| 8 | -2539.61 | 7.89 | 6.01 | -2566.47 | 6.56 | 7.25 | -2569.2 | 5.42 | 6.91 |
| 9 | -2550.8 | 6.22 | 6.03 | -2557.14 | 7.1 | 7 | -2525.51 | 6.5 | 6.51 |
| 10 | -2527.75 | 6.38 | 5.63 | -2549.98 | 5.2 | 7.8 | -2546.36 | 5.4 | 7.82 |
| Ave. | -2553.322 | 7.082 | 6.941 | -2549.204 | 6.049 | 7.441 | -2555.569 | 5.891 | 6.952 |
| S.E.M. | 4.880252821 | 0.263972726 | 0.401224252 | 4.63248469 | 0.288857924 | 0.166008735 | 4.037471845 | 0.239037445 | 0.288481542 |

## Slide 4
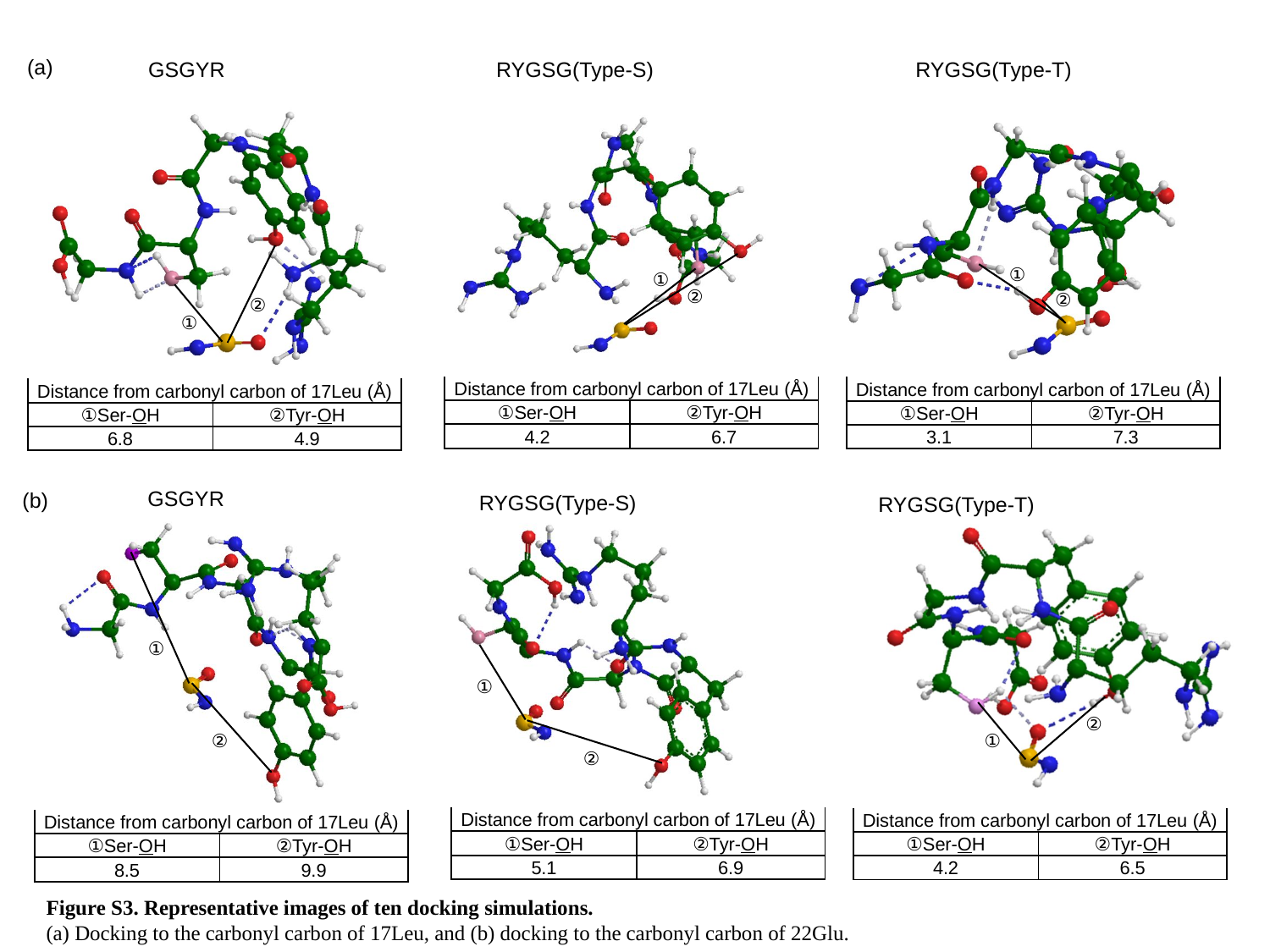

(a)
GSGYR
RYGSG(Type-S)
RYGSG(Type-T)
①
①
②
②
②
①
| Distance from carbonyl carbon of 17Leu (Å) | |
| --- | --- |
| ①Ser-OH | ②Tyr-OH |
| 4.2 | 6.7 |
| Distance from carbonyl carbon of 17Leu (Å) | |
| --- | --- |
| ①Ser-OH | ②Tyr-OH |
| 3.1 | 7.3 |
| Distance from carbonyl carbon of 17Leu (Å) | |
| --- | --- |
| ①Ser-OH | ②Tyr-OH |
| 6.8 | 4.9 |
GSGYR
(b)
RYGSG(Type-S)
RYGSG(Type-T)
①
①
②
①
②
②
| Distance from carbonyl carbon of 17Leu (Å) | |
| --- | --- |
| ①Ser-OH | ②Tyr-OH |
| 5.1 | 6.9 |
| Distance from carbonyl carbon of 17Leu (Å) | |
| --- | --- |
| ①Ser-OH | ②Tyr-OH |
| 4.2 | 6.5 |
| Distance from carbonyl carbon of 17Leu (Å) | |
| --- | --- |
| ①Ser-OH | ②Tyr-OH |
| 8.5 | 9.9 |
Figure S3. Representative images of ten docking simulations.
(a) Docking to the carbonyl carbon of 17Leu, and (b) docking to the carbonyl carbon of 22Glu.
